# The future of postgraduate training

**DOI:** 10.11604/pamj.2014.19.333.5555

**Published:** 2014-11-27

**Authors:** Kieran Walsh

**Affiliations:** 1 Clinical director, BMJ Learning, BMJ Publishing Group, BMA House, Tavistock Square, London WC1H 9JR, United Kingdom

**Keywords:** Postgraduate medical education, future, patients

## Abstract

Improvements to postgraduate training have included newly designed postgraduate curricula, new forms of delivery of learning, more valid and reliable assessments, and more rigorous evaluation of training programmes. All these changes have been necessary and have now started to settle in. Now therefore is an appropriate time to look to the future of postgraduate training. Predicting the future is difficult in any course of life-however an examination of recent trends is often a good place to start. In this regard the recent trend to start to produce more doctors and healthcare professionals of the type that the population needs is likely to continue for some time to come. Medical education will also need to be more flexible in the future. The more flexible that training programmes are, the more likely that we will have experts that are sufficiently flexible to meet a range of different challenges throughout the rest of their careers. Medical education will also become more seamless in the future (at present there are probably too many major milestones and transitions in medical education). In the future educators will make much more use of technology enhanced learning, e-learning and simulation in postgraduate medical education. There will also be more pressure on postgraduate training programmes to offer value for money and to be able to demonstrate such value for money. Postgraduate medical education of the future will also be a more personalised and adaptive experience. It will be far more based on learners’ individual needs and will be more responsive to those needs. Lastly postgraduate education will be much more closely supervised than it has been in the past. A common theme running through these changes will be patient centredness. This will mean safer training programmes that produce the type of doctors that patients and populations need.

## Editorial

Postgraduate training in medicine has undergone significant changes over the past thirty years. In many specialties postgraduate training hardly existed thirty years ago and in many others it was based largely on an apprenticeship model where newly qualified doctors gained experience in a haphazard and largely unsupervised way. Improvements have included newly designed postgraduate curricula, new forms of delivery of learning, more valid and reliable assessments, and more rigorous evaluation of training programmes [1, 2]. All these changes have undoubtedly been necessary and have now started to settle in. Now therefore is likely to be an appropriate time to look to the future of postgraduate training.

Predicting the future is difficult in any course of life-however an examination of recent trends is often a good place to start. In this regard the recent trend to start to produce more doctors and healthcare professionals of the type that the population needs is likely to continue for some time to come. In the past postgraduate training has sometimes appeared as if it was designed to meet the needs of teaching hospitals or to replace specialists that are already in post-rather than to produce specialists and generalists of the type that the disease burden in the population at large renders necessary. For example increased numbers of elderly people with multiple comorbidities should generate increased training programmes in geriatric medicine and general practice. While specialists will always be needed, the large epidemiological changes that are now happening will mean that the future will be in broad based training programmes that will ultimately produce generalists.

Medical education will also need to be more flexible in the future. It takes up to fifteen years to bring an entrant into medicine up to the standard of that of a fully qualified expert and in that time there will be many changes in medicine and in the expectations of trainees. For that reason the more flexible that training programmes are, the more likely that we will have experts that are sufficiently flexible to meet a range of different challenges throughout the rest of their careers. Until now postgraduate training has been at times remarkably inflexible. For example trainees who have wished to change career pathways have been forced to start again at the beginning of a new pathway without any of their previous experience being recognised. Trainees of the future will want to have a better work life balance and so the capability to step in and out training programmes will increasingly be necessary and indeed appreciated by trainees. Medical education will thus proceed at a pace that suits the learner-in some cases it will take longer than the specified time to achieve competency and in other cases it will take less time.

Medical education will also become more seamless in the future. At present there are probably too many major milestones and transitions in medical education [3]. In the future medical students should have sufficient experience to be able to start as doctors with far fewer challenges. At the other end of training there will be more recognition that, even though formal training programmes must come to an end, training continues throughout a doctor's career [4]. In the future educators will make much more use of technology enhanced learning, e-learning and simulation in postgraduate medical education [5, 6]. Working time restrictions will mean that postgraduate trainees will spend less time learning while working and this will be an important factor in encouraging the take up of more technology enhanced learning [7]. Other factors that will drive the uptake of technology enhanced learning will include the need for safe training environments, the need for trainees to avoid having to practice on real patients, and the need for trainees to be able to demonstrate their competence in assessment scenarios that are close to real life.

In the future there will be more pressure on postgraduate training programmes to offer value for money and to be able to demonstrate such value for money [8, 9]. Postgraduate medical education is expensive but until now there has been precious little attention paid to the cost of this form of education [10]. There have been some small recent studies in this regard that have looked at components of medical education but it is likely that in the future such studies will be more comprehensive, programmatic and prospective [11]. The funding constraints on healthcare in general and on healthcare professional education in particular will continue for some time to come and postgraduate education will not escape scrutiny. If the past twenty years have been about developing more effective methods of medical education, then the next thirty will probably be about developing more cost effective methods.

Postgraduate medical education of the future will also be a more personalised and adaptive experience. It will be far more based on learners’ individual needs and will be responsive to those needs in a way that has been impossible up to now [12]. These needs will span the professional competencies that are required to be a good doctor-including applied knowledge, communication and clinical skills, healthy attitudes, and professional behaviours. Continuous progress testing against required standards in all these fields will give individual learners and their educators a deep insight into where they are in their learning and the challenges that lie ahead of them. Lastly postgraduate education of the future will be much more closely supervised than it has been in the past [13]. There will be closer clinical and education supervision. This will ensure a better educational experience for learners and more importantly safer clinical care for patients who are being managed by doctors in training. The closest supervision will be targeted at those who need it most-that is, newly graduated doctors in their first two years of clinical work [14]. As postgraduate trainees become more experienced, supervision will gradually be loosened. By the time that trainees are nearly ready to be signed off as specialists, they will be working almost autonomously within their sphere of developing expertise. There will undoubtedly be other changes to postgraduate medical education but a common theme running through all of them will be patient centredness. This will ultimately mean safer training programmes that produce the type of doctors that patients and populations need.
